# Upregulation of a MicroRNA Signature Involving miR-17-5p, miR-26b-5p, miR-106a-5p, and miR-146a-5p During Cervical Epithelial Transformation

**DOI:** 10.3390/epigenomes10010001

**Published:** 2025-12-26

**Authors:** Andrea Hornakova, Zuzana Kolkova, Lucia Kotulova, Tomas Rokos, Ivana Kasubova, Terezia Pribulova, Erik Kozubik, Kamil Biringer, Erik Kudela, Veronika Holubekova

**Affiliations:** 1 Laboratory of Genomics and Prenatal Diagnostics, Biomedical Centre Martin, Jessenius Faculty of Medicine in Martin, Comenius University Bratislava, 03601 Martin, Slovakia; andrea.hornakova@uniba.sk (A.H.); 1uzana.snahnicanova@uniba.sk (Z.K.); ivana.kasubova@uniba.sk (I.K.); veronika.holubekova@uniba.sk (V.H.); 2 Laboratory of Bioinformatics and Biostatistics, Biomedical Centre Martin, Jessenius Faculty of Medicine in Martin, Comenius University Bratislava, 03601 Martin, Slovakia; lucia.kotulova@uniba.sk; 3 Clinic of Gynaecology and Obstetrics, Jessenius Faculty of Medicine in Martin, Comenius University Bratislava, 03601 Martin, Slovakia; rokos1@uniba.sk (T.R.); vlcakova8@uniba.sk (T.P.); erik.kozubik@uniba.sk (E.K.); kamil.biringer@uniba.sk (K.B.); erik.kudela@uniba.sk (E.K.)

**Keywords:** cervical cancer, miRNA expression, HPV infection, signalling pathways

## Abstract

**Background**: Cervical cancer remains the fourth most common malignancy among women worldwide. Despite vaccination and regular screening, new molecular biomarkers are needed for improved early detection and risk assessment. MicroRNAs (miRNAs) play crucial roles in post-transcriptional regulation, and their dysregulation may contribute to cervical carcinogenesis. This study evaluated the expression of selected miRNAs in cervical swab samples and corresponding biopsies from women with various grades of cervical lesions and assessed their relationship with human papillomavirus (HPV) infection. **Methods**: A total of 72 cervical swab samples were included in this study, divided according to cytological severity: negative for intraepithelial lesion or malignancy (NILM, *n* = 15), atypical squamous cells of undetermined significance (ASC-US, *n* = 12), low-grade squamous intraepithelial lesion (LSIL, *n* = 19), and high-grade squamous intraepithelial lesion (HSIL, *n* = 26). In a subset of patients, corresponding biopsy specimens were analysed for comparison. The association of miRNA expression with HPV infection status was also examined. miRNA expression was quantified by real-time PCR using commercially available assays. **Results**: To assess the relationship between miRNA expression, lesion severity, and HPV infection, fold change values were compared to the control group (NILM). No significant differences were observed in the ASC-US group (*p* > 0.05). In contrast, several miRNAs were significantly upregulated in the LSIL and/or HSIL groups, as well as in HPV-positive samples, indicating their association with both lesion progression and viral infection. Specifically, miR-17-5p, miR-26b-5p, miR-29a-3p, miR-103a-3p, miR-106a-5p, miR-146a-5p, miR-155-5p, and miR-191-5p showed increased expression (*p* < 0.05) compared with controls. The observed upregulation of miR-26b-5p, miR-106a-5p, and miR-146a-5p highlights their potential role in HPV-associated cervical carcinogenesis. Dysregulated miRNAs were enriched in pathways related to infectious diseases, various types of cancer, and cell adhesion processes. **Conclusions**: The gradual increase in specific miRNAs with lesion severity and HPV infection suggests their role in cervical carcinogenesis. The identified miRNAs may serve as promising non-invasive biomarkers for early detection and monitoring of HPV-associated cervical lesions.

## 1. Introduction

Cervical cancer (CC) is a malignant tumour of the cervix, most commonly caused by persistent infection with high-risk types of human papillomavirus (HPV). More than 200 HPV types have been identified, with types 16 and 18 accounting for approximately 70% of all CC cases worldwide [1]. CC remains the fourth most common cancer in women in terms of both incidence and mortality, with an estimated 660,000 new cases and 350,000 deaths globally in 2022 [2]. Early detection of CC is crucial, as delayed diagnosis significantly reduces survival rates in women worldwide. In its early stages, CC is often asymptomatic, which complicates timely detection and diagnosis. In this context, the identification of reliable tumour biomarkers plays a vital role. Tumour biomarkers, molecules detectable in body fluids or tissues, are frequently associated with cancer presence, progression, or prognosis. These include genes, DNA, RNA, microRNAs (miRNAs), proteins, and other biological products [3]. Detecting such biomarkers in the early stages of carcinogenesis can provide essential information for screening, diagnosis, and prognosis prediction. Moreover, biomarker profiling enables the assessment of disease extent, monitoring of treatment response, and development of personalised therapeutic strategies [4]. In recent years, considerable efforts have been devoted to identifying specific biomarkers for CC and translating these findings into clinical practice, offering new opportunities for improving early detection and patient outcomes.

**Figure 1:**
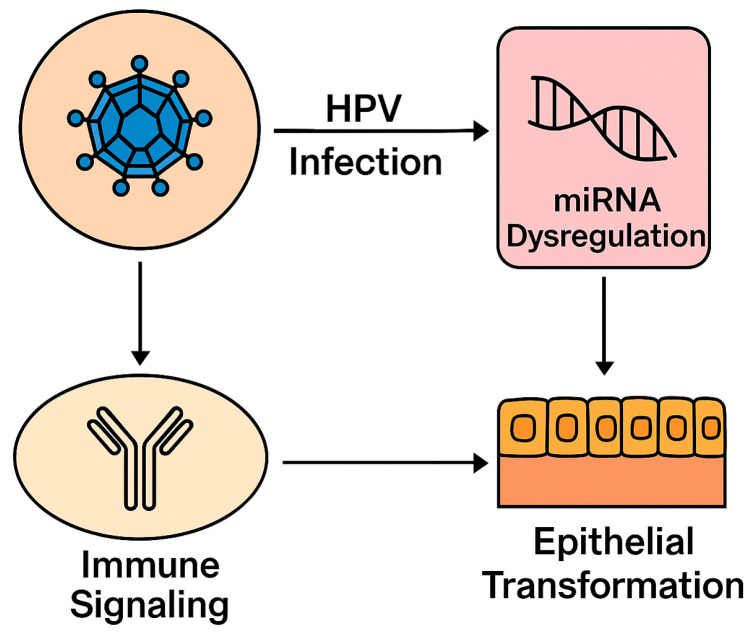
Conceptual diagram of HPV-induced miRNA dysregulation, immune signalling interference, and epithelial transformation. Figure created by the authors using AI-assisted illustration (OpenAI).

MicroRNAs (miRNAs) are small, non-coding RNA molecules averaging approximately 22 nucleotides in length. They are initially transcribed from DNA into primary miRNAs (pri-miRNAs), processed into precursor miRNAs (pre-miRNAs), and finally cleaved into mature miRNAs. Most miRNAs regulate gene expression post-transcriptionally by binding to complementary sequences within the 3′ untranslated region (3′ UTR) of target messenger RNAs (mRNAs), resulting in mRNA degradation or translational repression [5]. miRNAs play an essential role in numerous biological processes, including cell proliferation, differentiation, and apoptosis [6]. Aberrant miRNA expression has been reported in many malignancies, including CC, suggesting that dysregulation of specific miRNAs may contribute to tumour initiation, progression, and metastasis. MiRNAs can act as oncogenes, promoting tumour development, or tumour suppressors, inhibiting cancer-related pathways [7]. A distinct miRNA expression profile has been associated with different stages of cervical neoplasia, HPV status, and patient prognosis. Certain miRNAs have also been proposed as promising non-invasive biomarkers for early detection and as targets for precision therapies, particularly for overcoming chemoresistance [8]. The conceptual model illustrating how HPV infection orchestrates miRNA dysregulation, immune signalling, and epithelial transformation is displayed in Figure 1.

Furthermore, miRNAs are secreted into extracellular fluids such as blood, urine, and cervical secretions, making them accessible and minimally invasive biomarkers for cancer detection and monitoring. Beyond their biomarker potential, miRNAs also participate in intercellular communication and signalling [9,10].

In this study, we analysed the expression profiles of selected miRNAs in cervical swab samples representing various diagnostic categories, including negative for intraepithelial lesion or malignancy (NILM), atypical squamous cells of undetermined significance (ASC-US), low-grade squamous intraepithelial lesion (LSIL), high-grade squamous intraepithelial lesion (HSIL), and CC. Our primary objective was to evaluate the role of specific miRNAs in cervical carcinogenesis and to assess their potential as diagnostic or prognostic biomarkers. Understanding the complex regulatory networks involving miRNAs in HPV-associated carcinogenesis may contribute to improved disease classification, early detection, and the development of personalised therapeutic strategies.

We hope that our findings will deepen the understanding of miRNA involvement in CC pathogenesis and support their future integration into clinical diagnostics and targeted therapeutic strategies.

## 2. Results

### 2.1. miRNA Expression Among Cytological Lesion Grades

To evaluate differences in miRNA expression among cervical lesion grades, fold change (FC) values were compared to the control group (NILM). As shown in Table 1, several miRNAs exhibited increased expression with the severity of lesions. In the ASC-US group, none of the analysed miRNAs showed a significant difference in expression compared with controls (*p* > 0.05). In contrast, we observed significant upregulation for several miRNAs in the LSIL and HSIL groups, indicating an association between miRNA dysregulation and lesion severity. Specifically, miR-17-5p, miR-26b-5p, miR-29a-3p, miR-103a-3p, miR-106a-5p, miR-146a-5p, miR-155-5p, and miR-191-5p displayed significant overexpression (*p* < 0.05) in the LSIL and/or HSIL groups compared with controls. Among these, miR-26b-5p, miR-103a-3p, and miR-146a-5p showed the most pronounced fold changes—miR-26b-5p and miR-146a-5p exhibited up to a fourfold increase in expression in the LSIL and/or HSIL groups; miR-106a-3p showed nearly a fivefold increase in the HSIL group compared to the control group.

Overall, the expression patterns revealed a gradual increase in several miRNAs from low-grade to high-grade lesions, supporting their potential role in cervical carcinogenesis and their applicability as non-invasive biomarkers for disease progression.

**Figure 2:**
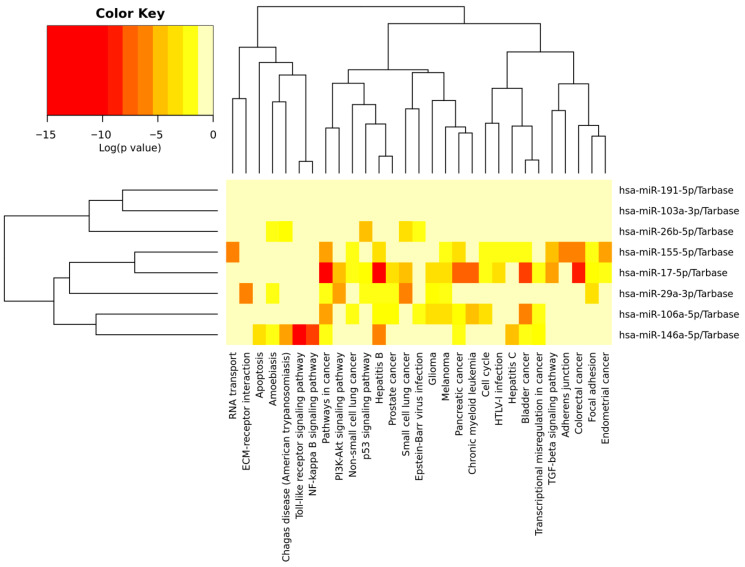
Heatmap displaying the enrichment of deregulated miRNAs in cytological lesions in KEGG signalling pathways. The significance of miRs in signalling pathways is represented by the intensity of the red colour (redder indicates a more significant result).

Eight of the differentially expressed miRNAs were analysed using the KEGG Pathway database. Five deregulated miRNAs were involved in cancer pathways, specifically bladder, colorectal, pancreatic, prostate, and endometrial cancer, as well as small and non-small cell lung cancer, leukaemia, melanoma, and glioma. The deregulated miRNAs mostly influenced signalling pathways associated with immune response and inflammation (toll-like receptor and NF-kappa B signalling pathways) and with cell survival, growth, proliferation, and the cell cycle (PIK3-Akt, TGF-beta, p53, and NF-kappa B signalling pathways). Abnormalities in miRNA gene expression lead to changes in cellular adhesion, such as focal adhesion, adherens junctions–ECM receptor interaction, cell cycle and transcription misregulation, RNA transport, and apoptosis. The signalling pathways, along with corresponding *p*-values and dysregulated genes, are listed in Supplementary File S1. The detailed functions of deregulated miRNA are displayed in Figure 2.

### 2.2. Association Between HPV Infection and Deregulated miRNAs

To investigate the effect of HPV infection on miRNA expression, FC values were compared between HPV-positive individuals and controls (Table 2). Overall, several miRNAs showed increased expression levels in HPV-positive samples. Among the analysed miRNAs, miR-26b-5p and miR-146a-5p were significantly upregulated in the HPV-positive group (*p* < 0.05). Especially, miR-26b-5p exhibited the most pronounced increase in expression (FC = 2.62), followed by miR-146a-5p (FC = 2.27), indicating their strong association with HPV infection. Other miRNAs, such as miR-32-5p, miR-106a-5p, and miR-148b-3p, showed a trend toward upregulation (*p* < 0.1), suggesting possible HPV-related modulation that did not reach statistical significance.

**Figure 3:**
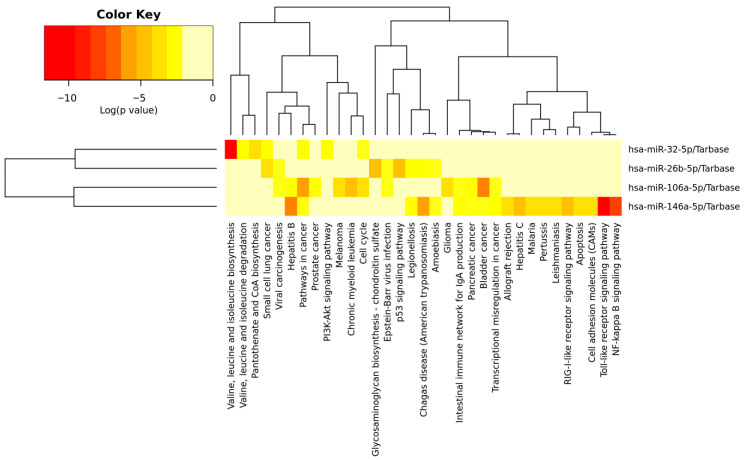
Heatmap displaying the enrichment of deregulated miRNAs in HPV infected cervical lesions in KEGG signalling pathway analysis. The significance of miRs in signalling pathways is represented by the intensity of the red colour (redder means a more significant result).

The analysis of signalling pathways indicates that HPV infection influences the immune response to viral infection, including toll-like receptor, NF-kappa B, RIG-I-like receptor, allograft rejection, and the intestinal immune network for IgA production. Significantly deregulated miRNAs were also involved in cancer pathways, specifically bladder cancer, small cell lung cancer, chronic myeloid leukaemia, prostate cancer, and pancreatic cancer, as well as melanoma and glioma. The analysed miRNAs are also enriched in infectious diseases, such as hepatitis B, Chagas disease, legionellosis, Epstein–Barr virus infection, hepatitis C, amoebiasis, malaria, pertussis, and leishmaniasis. Deregulated miRNAs also influenced metabolic pathways, such as valine, leucine, and isoleucine biosynthesis and degradation; glycosaminoglycan biosynthesis (chondroitin sulphate and pantothenate); and CoA biosynthesis. Other deregulated pathways were cell cycle, viral carcinogenesis, and transcriptional misregulation in cancer; cell adhesion molecules; and apoptosis, in which the p53 and PI3K-Akt signalling pathways are involved. The signalling pathways, along with corresponding *p*-values and dysregulated genes, are listed in Supplementary File S1. The detailed functions of deregulated miRNA are displayed in a heatmap in Figure 3.

These results suggest that HPV infection contributes to altered miRNA expression, particularly affecting miRNAs previously implicated in cell proliferation, apoptosis, and immune regulation. The observed upregulation of miR-26b-5p, miR-106a-5p, and miR-146a-5p highlights their potential role in HPV-associated cervical carcinogenesis.

### 2.3. Different Expression of miRNAs in Cervical Lesion Biopsies

Analysis of the expression of our monitored miRNAs across cervical lesion grades revealed distinct patterns of dysregulation associated with the progression of cervical intraepithelial neoplasia. Several miRNAs showed statistically significant increases in expression across CIN grades (listed in Table 3).

In particular, a significant upregulation of miR-26b-5p, miR-103a-3p and miR-106a-5p was detected in the CIN2 group (*p* = 0.01, *p* = 0.005, and *p* = 0.005, respectively), suggesting their involvement in early stages of cervical lesion development.

**Figure 4:**
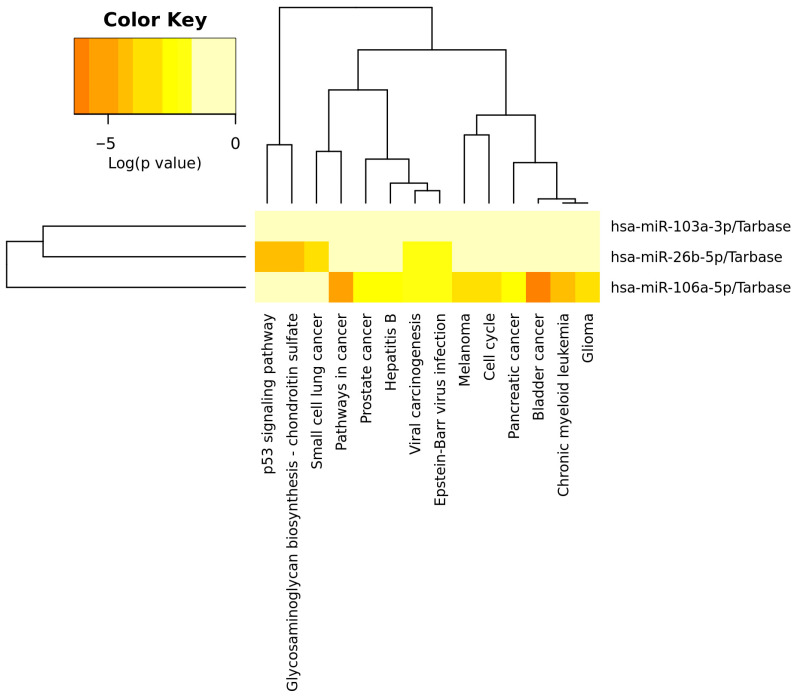
Heatmap displaying the enrichment of deregulated miRNAs in worsening biopsies in KEGG signalling pathway analysis. The significance of miRs in signalling pathways is represented by the intensity of the red colour (redder means a more significant result).

The significantly upregulated miRNAs were analysed using the KEGG Pathway database. The most enriched pathways were related to cancer pathways, such as bladder cancer, small cell lung cancer, chronic myeloid leukaemia, glioma, melanoma, prostate cancer, and pancreatic cancer. The development of cancer is associated with abnormal expression in the p53 signalling pathway, cell cycle, and viral carcinogenesis, where the most dysregulated genes are influenced by miR-26b-5p. The deregulated miR-26b-5p also influences genes involved in glycosaminoglycan biosynthesis—the chondroitin sulphate signalling pathway. The signalling pathways, along with corresponding *p*-values and dysregulated genes, are listed in Supplementary File S1. The detailed functions of deregulated miRNA are displayed in Figure 4.

These miRNAs (miR-26b-5p, miR-103a-3p, and miR-106a-5p) are known to regulate genes implicated in cell proliferation and apoptosis [11,12,13], indicating that their altered expression may contribute to the disruption of cellular homeostasis.

Moreover, miR-155-5p and miR-484 were significantly upregulated in the CIN3 group (*p* = 0.004 and *p* = 0.048, respectively), consistent with their previously reported role in inflammation and oncogenesis. MiR-155-5p has a complex role in microbial infections, acting as a key regulator of the immune response [14,15]. In the most advanced group (CIN3-CIS), a significant increase in miR-103a-3p (*p* = 0.048) was observed, suggesting its potential involvement in the transition from high-grade dysplasia to carcinoma in situ.

Although miR-191-5p exhibited moderate fold changes, these differences did not reach statistical significance.

The identified miRNAs, particularly miR-26b-5p, miR-103a-3p, miR-106a-5p, miR-155-5p, and miR-484, are promising candidates for further investigation as potential biomarkers of cervical lesion progression.

## 3. Discussion

This study’s results revealed the involvement of significantly deregulated miRNAs in signalling pathways that mediate host cell responses to infectious diseases through the immune response, as well as pathways that lead to cancer development.

Specific miRNAs are altered in both tumour tissues and the circulation of cancer patients [16], including patients with CC, highlighting their potential as non-invasive biomarkers for diagnosis and disease monitoring [10,17,18].

Dysregulation of miRNAs, particularly those involved in cell cycle and apoptosis control, contributes to HPV-mediated carcinogenesis [5,13,19].

Previous studies have shown that miRNA-106a-5p acts as an oncogenic miRNA (oncomiR) and is significantly upregulated in CC tissues and cell lines. Its increased expression correlates with HPV positivity and enhanced cell migration and invasion [13,20,21]. High-risk HPV types (HPV16/18) modulate miR-106a through E6/E7 oncoprotein-mediated disruption of miRNA biogenesis enzymes Drosha and Dicer and interference with the p53/pRb pathways [22]. In our cohort, miR-106a-5p was markedly (nearly fivefold) elevated in LSIL and HSIL samples, CIN2 biopsies, and HPV-positive cases, supporting its role in early cervical carcinogenesis. Functionally, miR-106a targets tumour-suppressive genes such as tissue inhibitor of metalloproteinases 2 (TIMP2), which normally prevents extracellular matrix degradation and liver kinase B1 (LKB1), thereby promoting degradation of the extracellular matrix and activation of the PI3K/AKT and AMPK-mTOR pathways. Through these mechanisms, miR-106a enhances the proliferation, migration, and invasion of CC cells. These findings highlight miR-106a-5p as a potential biomarker and therapeutic target in HPV-related cervical lesions [13,21].

Similarly, members of the miR-17~92 cluster, particularly miR-17-5p, promote tumorigenesis by enhancing proliferation and inhibiting apoptosis. miR-17-5p acts as an oncomiR and serves as a key regulator of proliferation, autophagy, and apoptosis [23]. Overexpression of miR-17-5p has been reported in multiple cancers, including lung [24], breast [25], colorectal [26], prostate [27], and CC [28,29], where it enhances cell proliferation, migration, and invasion by targeting transforming growth-factor-β receptor 2 (TGFBR2) and TIMP2, facilitating extracellular matrix degradation and epithelial–mesenchymal transition [28,30]. Genetic variants within the miR-17-5p gene (rs217727, rs2862833, rs9931702, and rs9302648) have also been associated with increased susceptibility to CC [29]. In our study, we observed significantly increased levels of miR-17-5p with cervical lesion severity and HPV positivity, although the differences did not reach statistical significance. These findings support the link to HPV-driven miR-17-5p dysregulation and promote cervical epithelial transformation. However, other studies suggest a potential tumour-suppressive role by targeting CDC10-dependent transcript-2 (Cdt2), leading to S-phase arrest and reduced proliferation, migration, and invasion of CC cells, while non-cancerous cells remain unaffected [31]. This dual behaviour suggests a context-dependent function as an oncogene or tumour suppressor depending on the cellular background.

The interplay between oncogenic and tumour-suppressive miRNAs reflects the complexity of carcinogenesis. In contrast, miR-26b-5p acts mainly as a tumour suppressor, regulating proliferation, migration, invasion, and apoptosis. Conversely, its downregulation in breast cancer [32] and/or osteosarcoma leads to increased levels of connective tissue growth factor (CTGF) and Smad1, promoting metastasis [33]. Similarly, its downregulation in CC tissues and cell lines correlates with more aggressive tumour features. Functionally, miR-26b-5p inhibits cell migration and invasion through JAG1 or PI3K/AKT modulation [34,35]. In addition, six miRNAs (miR-26b-5p, miR-146b-5p, miR-191-5p, miR-484, miR-574-3p, miR-625-3p) have been identified to be associated with CIN and CC [11]. Our results showed significantly higher miR-26b-5p expression in both the LSIL and HSIL groups and HPV-positive patients, which contrasts with previous reports of its downregulation in invasive CC. This upregulation in precancerous lesions may reflect an early host response to HPV infection, potentially aimed at limiting viral replication or oncogenic transformation. Taken together, miR-26b-5p plays a predominantly tumour-suppressive role across various cancers, including CC. While its downregulation promotes proliferation, invasion, and metastasis, our results suggest that miR-26b-5p is upregulated in precancerous cervical lesions, particularly in HPV-positive patients. This upregulation may serve as an early defence against HPV-induced oncogenesis. The divergent expression patterns highlight the context-dependent regulation of miR-26b-5p during cervical carcinogenesis, warranting further research to clarify its role as a biomarker and therapeutic target in HPV-related cervical disease.

The dynamic and context-dependent regulation of miRNAs, such as miR-26b-5p, highlights the complexity of post-transcriptional control in cervical lesion development. While miR-26b-5p acts mainly as a tumour suppressor, other miRNAs, like miR-32-5p, show dual regulatory roles in various cancers. For example, miR-32-5p inhibits migration and invasion in non-small cell lung cancer (NSCLC) by targeting SMAD3 and modulating the TGF-β/SMAD signalling pathway, which is crucial for tumour progression and immune regulation [36]. Although its role in HPV-related cervical carcinogenesis is not fully understood, the TGF-β/SMAD and PI3K/AKT pathways, both affected by HPV oncoproteins E6 and E7, are known to be dysregulated during HPV infection [37,38].

Higher levels of miR-32-5p were also found in both serum and tissue samples from CC patients [39]. Another study found significantly decreased expression of miR-32-5p in CC tissues and cell lines, where it inhibited malignant cell behaviour by targeting HOXB8 [40]. In our cohort, we observed borderline expression of miR-32-5p in HPV-positive samples, suggesting its potential association with viral infection-related molecular events. This may indicate that miR-32-5p is involved in the host cellular response to HPV—altered miR-32-5p expression could influence immune modulation, epithelial differentiation, or viral persistence, highlighting its potential as a novel biomarker or regulatory molecule in HPV-associated lesions.

Similarly to previous miRNAs, miR-146a-5p exhibits context-dependent functions, acting as both a tumour suppressor and an oncogene across different malignancies. In triple-negative breast cancer [41] and prostate cancer [42], miR-146a-5p has been described as a tumour suppressor that inhibits cell proliferation, migration, and invasion. The restoration of miR-146a-5p expression significantly inhibited proliferation and metastasis by suppressing SOX5 [41]. In contrast, miR-146a-5p acts as an oncogene in gastric cancer [43]. In NSCLC, its expression varies, depending on the biological sample. In NSCLC cell lines, it is downregulated with a tumour-suppressive effect [44], while in both serum and tumour tissues, it is overexpressed [45]. Although elevated miR-146a-5p expression has been observed in CC, its role as a tumour suppressor remains unclear [46]. Our study showed increased miR-146a-5p expression in both the LSIL and HSIL groups, as well as in HPV-positive samples. These findings suggest that miR-146a-5p may play a regulatory role in the early stages of cervical lesion development. Its expression could be modulated by HPV infection, supporting its potential as a tumour-suppressive factor in HPV-driven transformation. Further research is needed to identify its downstream targets and evaluate its potential as a diagnostic biomarker or therapeutic target.

HPV infection influences the expression of multiple miRNAs involved in inflammation, immune regulation, and epithelial homeostasis. The miR-29 family, including miR-29a-3p, miR-29b-1, miR-29b-2, and miR-29c, plays an important role in maintaining normal cell differentiation, proliferation, and apoptosis [47]. Its downregulation in CC indicates its tumour-suppressive function [48] and might be linked to tumour cell proliferation, invasion, and epithelial–mesenchymal transition. Mechanistically, miR-29a-3p inhibits oncogenic molecules, such as CDC42, DNMT3A, and DNMT3B [48].

Contrary to previous studies, our results show increased expression of miR-29a-3p in LSIL and HSIL samples, suggesting a compensatory response in early stages of epithelial transformation rather than in invasive cancer progression. Dysregulation of miRNA expression during cervical carcinogenesis depends on the cellular environment, HPV infection, and lesion stage. There is a lack of studies on miRNA expression during cervical lesion progression; therefore, we suggest that the process of cervical epithelial transformation depends on the dynamic balance between tumour-suppressive and adaptive cellular mechanisms during lesion progression.

While miR-29a-3p primarily acts as a tumour suppressor during the early phases of cervical epithelial transformation, another miRNA, miR-155, represents one of the best-characterised oncomiRs and is linked to enhanced proliferation, invasion, and immune modulation in various cancers, including CC [49,50]. Its upregulation correlates with advanced disease and poor prognosis, promoting tumour progression by modulating cell proliferation, migration, invasion, and the cell cycle, and is also linked to drug resistance, while its inhibition may restore chemosensitivity [51]. We observed significant upregulation of miR-155-5p in HSIL and CIN3 lesions, consistent with previous studies on cancer tissues [51,52,53] and cervical scrapes or urine samples [54]. Furthermore, miR-155 overexpression was present during the transition from CIN1 to squamous cell carcinoma, with a statistically significant elevation in the CIN2–3 group compared with the CIN group, further supporting its association with lesion progression [55]. These observations collectively support the potential of miR-155 as a non-invasive biomarker for cervical disease progression. Elevated miR-155 expression has also been associated with HR-HPV infection, suggesting its role in HPV-driven carcinogenesis by targeting TP53INP1 in the TP53 signalling pathway [56]. Overall, our results and previously published data demonstrate that miR-155 overexpression accompanies the progression of cervical lesions, supporting its potential as a non-invasive biomarker in CC diagnostics, prognostics, and therapeutics.

On the other hand, dysregulation of miR-103a-3p has also been reported in various cancers. In glioma and NSCLC, miR-103a-3p was found to be downregulated, which exerted antiproliferative and pro-apoptotic effects, suggesting a tumour-suppressive function [57]. On the contrary, several studies have demonstrated miR-103a-3p upregulation in neuroblastoma, gastric cancer, breast cancer [58,59], and colorectal cancer [60], promoting tumour growth, migration, and invasion. On the other hand, miR-103a-3p has shown inconsistent expression in CC. It was upregulated in CC tissues and cell lines, promoting proliferation and suppressing apoptosis [12]. Another study reported reduced miR-103a-3p expression in CC cells, suggesting that its regulatory effect may depend on the cellular context or on specific molecular interactions [57]. Our study found significant miR-103a-3p upregulation in LSIL, HSIL, CIN2, and CIN3 samples, suggesting its involvement in early and intermediate stages of epithelial transformation.

### Clinical Implications

The gradual upregulation of several miRNAs—particularly miR-26b-5p, miR-106a-5p, and miR-146a-5p—in LSIL and HSIL, as well as in HPV-positive samples, suggests their potential utility in refining risk differentiation among HPV-positive women. Since these miRNAs showed no significant changes in ASC-US but were elevated in more advanced lesions, they may in future help identify patients with a higher likelihood of clinically relevant disease and support more informed decisions regarding referral for colposcopy. Although further validation and functional studies are required, our findings indicate that the assessed miRNAs could serve as complementary markers contributing to improved risk stratification in the clinical management of HPV-positive patients.

## 4. Materials and Methods

### 4.1. Study Population

Patients with long-term cervical abnormalities were referred by their local gynaecologists to the Clinic of Expert Colposcopy at the Clinic of Gynaecology and Obstetrics, Jessenius Faculty of Medicine, Comenius University in Bratislava and University Hospital Martin, as well as patients with normal findings who were examined at the clinic for another diagnosis. All participants were informed about the study and signed the informed consent approved by the Ethics Committee of the Jessenius Faculty of Medicine in Martin, Comenius University in Bratislava (Approval Codes EK85/2020 and EK05/2024).

Cervical samples were taken using nylon flocked swabs from the ectocervical area and posterior vaginal fornix during expert colposcopic examination. Cervical samples for molecular testing were immersed in viral/bacterial transport medium (Sample Preservation Solution, Mole Bioscience, Jiangsu, China) and transported to the Laboratory of Genomics and Prenatal Diagnostics, Biomedical Centre Martin, Jessenius Faculty of Medicine, Comenius University in Bratislava, where the medium was aliquoted and stored at −80 °C until the molecular analysis.

A total of 72 samples were collected from women with a median age of 39 years (age range 19 to 77 years) who were being followed up by a general gynaecologist for persistent abnormal cytology. Inclusion criteria were no pregnancy, no previous cervical treatments or hysterectomy, no immunosuppression, and persistent abnormal cytology. Patients’ characteristics are listed in Table 4.

Samples were categorised according to the severity of cytological abnormality based on the Bethesda classification [61]: NILM (*n* = 15; control group), ASC-US (*n* = 12), LSIL (*n* = 19), and HSIL (*n* = 26). Corresponding biopsy results were available for 9 patients in the ASC-US group, 17 in the LSIL group, and 24 in the HSIL group. No biopsies were performed in the NILM group.

The presence of HPV infection was determined by the method we previously described [62]. HPV genotypes were identified by PCR targeting the conserved L1 region of HPV-16 and -18, similar to the approach published by Gravitt et al. [63]. The study’s results did not affect standard diagnostic or clinical management procedures.

### 4.2. RNA Isolation and Reverse Transcription

Total RNA, including the miRNA fraction, was extracted from 500 μL of transport medium washed twice with PBS using the miRNeasy Micro Kit (Qiagen, Hilden, Germany), following the manufacturer’s protocol. The RNA was eluted in 25 μL of elution buffer. RNA concentration and quality were assessed using the Qubit^TM^ RNA HS Assay Kit on a Qubit^TM^ 3.0 Fluorometer (Thermo Fisher Scientific, Waltham, MA, USA), and RNA integrity was evaluated using an Agilent 2100 Bioanalyzer (Agilent Technologies, Santa Clara, CA, USA), with all samples showing RIN values ≥ 7.0. Isolated RNA was stored at −80 °C until further processing.

For reverse transcription, 1 μL of total RNA (depending on sample yield) was converted to complementary DNA (cDNA) using the miRCURY LNA RT Kit (Qiagen, Hilden, Germany) in 10 μL reaction volumes according to the manufacturer’s protocol. Each reaction mixture contained 2 μL of 5× miRCURY RT Reaction Buffer, 1 μL of 10× miRCURY RT Enzyme Mix, 0.5 μL of UniSp6 RNA and cel-miR-39 Spike-in mixture, RNase-free water, and 1 μL of total RNA. Reverse transcription was performed using the following cycling conditions: 42 °C for 60 min, followed by 95 °C for 5 min to inactivate the enzyme. cDNA samples were stored at −20 °C until downstream applications.

### 4.3. miRNA Expression Analysis

One microlitre of cDNA was diluted in a 1:30 ratio, and 3 μL of this dilution was added to each real-time PCR reaction, resulting in a total reaction volume of 10 μL. Reactions were performed in singleplex format using the miRCURY LNA miRNA PCR System (Qiagen, Hilden, Germany) with miRCURY LNA miRNA assays (Qiagen, Hilden, Germany), targeting both reference and target miRNAs (miR-16-5p, miR-21-5p, miR-103a-3p, miR-155-5p, miR-22-3p, miR-148a-3p, miR-191-5p, 146a-5p, miR-26b-5p, miR-106a-5p, miR-148b-3p, miR-484, miR-17-5p, miR-34a-3p, miR-29a-3p, miR-143-5p, miR-32-5p, and miR-423-3p). The panel of miRNAs was selected based on previous reports of their association with cervical lesion progression, HPV infection, and relevant biological pathways, such as cell proliferation, apoptosis, and immune response. Detailed information about the selected miRNAs is provided in Table 5. Each reaction was run in technical duplicates.

Quantitation of miRNAs and Cp value determination were performed using the second-derivative method on the LightCycler 480 (Roche Diagnostics GmbH, Mannheim, Germany) with the corresponding software. A Ct cut-off set 5 cycles before the end of the qPCR run (of the total 45 cycles) was applied for all miRNA assays, and assay efficiencies (90–110%, as provided by the manufacturer) were used to ensure accurate quantification. All qPCR runs included technical controls (no-template controls and no-reverse-transcription controls). Spike-in controls added during reverse transcription were used to monitor cDNA synthesis efficiency and inter-sample variability.

### 4.4. Cp-Value Analysis

The analysis of Cp values was performed using the GeneGlobe data analysis tool, available at https://geneglobe.qiagen.com/us/ (accessed on 3 October 2025). The tool allows for quality control of samples based on spike-ins added during RNA extraction and reverse transcription, in addition to data normalisation with the calculation of a stability factor for reference miRNAs, according to the geNorm algorithm (raw qPCR data are attached in Supplementary File S2).

As there are no universally accepted endogenous reference miRNAs, the stability of candidate reference genes was assessed using the geNorm algorithm available in the GeneGlobe tool. Based on this analysis, miR-16-5p and miR-21-5p were identified as the most stably expressed miRNAs across all control and experimental samples, with stability values of 0.1137 for miR-16-5p and 0.1190 for miR-21-5p, indicating low variability and high expression stability. These two miRNAs were subsequently used as housekeeping controls for normalisation of Cq values to ensure accurate quantification and minimise inter-sample variability. Normalised ∆Ct values were further log_2_-transformed for subsequent statistical analyses and visualisation. The average Cq values (arithmetic means) for normalised data were as follows: 23.59 for the control group, 23.30 for ASC-US, 24.00 for LSIL, and 23.21 for HSIL.

### 4.5. Statistical Analysis

The statistical analysis was performed in the R programming language (version 4.4.2) using the libraries cited in the References section [64,65,66,67,68,69]. Each gene (miRNA) was analysed separately. First, exploratory data analysis was performed, summarising medians and lower and upper quartiles for continuous variables and counts and percentages for factors; boxplots were used to visualise the distribution of FC across factor levels. For each miRNA and each clinical factor, an appropriate power transformation of the response (FC) was estimated using the powerTransform function. For each miRNA, separate linear models were then fitted, including age and a single clinical factor (biopsy, lesion, HPV, etc.) as predictors; interaction terms between clinical factors were not included because of sparse combinations of factor levels and the resulting instability of estimates. Undetectable or missing Ct values were treated as missing and excluded, so FC was calculated, and each model was fitted on complete cases for the given miRNA–factor combination. Model assumptions were assessed using Q–Q plots and histograms of residuals for normality, residuals versus fitted values for linearity and homoscedasticity, and Cook’s distances (and related influence measures) for influential observations. Post hoc analyses were performed using the emmeans package. The estimated marginal mean FC for each factor level was obtained from the fitted models and contrasted with the reference level (controls); pairwise comparisons between all factor levels were adjusted using Tukey’s method as implemented in emmeans. For each clinical factor, raw *p*-values from the primary contrasts (non-reference levels vs. controls) across all miRNAs and factor levels were additionally adjusted for multiple testing using the Benjamini–Hochberg false discovery rate (FDR) procedure. Summary tables (Table 1, Table 2 and Table 3) report estimated marginal means of FC together with 95% confidence intervals and *p*-values for testing whether the fold change differs from 1; in rare cases where model-based confidence intervals extended slightly below 0 due to high uncertainty and small numbers of observations, the lower confidence limit was truncated at 0 for presentation. R code implementing the main modelling steps (power transformation, linear models, post hoc analyses, and multiple-testing correction) is provided in the Supplementary File S3.

### 4.6. KEGG Enrichment Analysis of Differentially Expressed miRNA

The significantly expressed miRNAs with FDR-corrected *p*-values below 10% were also analysed using the mirPath v3 tool [70] to identify biological pathways in which the set of significantly deregulated miRNAs is enriched, and target genes were identified using TarBase v7.0. The analysis was set up for pathway union and FDR correction with a *p*-value threshold of 0.05. For rapid graphical visualisation of deregulated miRNAs in biological pathways, we used heatmaps; the complete results, including affected genes, are listed in Supplementary File S1.

## 5. Conclusions

In summary, this study demonstrates that specific miRNAs show consistent dysregulation across HPV-positive cervical lesions of increasing severity. The observed expression patterns and pathway analyses indicate that these miRNAs may serve as markers of early molecular changes associated with HPV infection and cervical epithelial alterations. The identified miRNAs represent promising candidates for future biomarker development, particularly for improving risk stratification and early detection.

Several limitations should be acknowledged, including the lack of detailed baseline characteristics (e.g., BMI, smoking status, hormonal status, parity, contraceptive use), the unequal distribution of diagnostic categories, the relatively small cohort size, and the absence of comprehensive HPV genotyping, which may limit generalisability.

Despite these limitations, the study has notable strengths. Minimally invasive cervical swab sampling, complemented by paired cytological and histological analyses, allowed practical assessment of miRNA expression. The reproducibility of miRNA signatures across lesion grades and their alignment with HPV status support the robustness of the findings.

Overall, this study enhances our understanding of miRNA dysregulation in cervical lesion progression and provides a foundation for further research. In particular, the consistent upregulation of miR-146-5p in high-grade lesions and HPV-positive samples highlights its potential involvement in cervical carcinogenesis through modulation of immune-related pathways, including the Toll-like receptor/NF-_k_B signalling pathway. Larger, longitudinal studies with functional validation are warranted to confirm the diagnostic and prognostic potential of these candidate miRNAs and to explore their integration into cervical screening strategies.

## Figures and Tables

**Table 1:** Expression changes in miRNAs across cervical lesions.

miRNAs	ASC-US (*n* = 12)		LSIL (*n* = 17)		HSIL (*n* = 25)		ct NILM (*n* = 15)
	*q Value* [*p Value*]	FC (95% CI)	*q Value* [*p Value*]	FC (95% CI)	*q Value* [*p Value*]	FC (95% CI)	(Mean ± SD)
miR-17-5p	0.93 [0.90]	1.03 (0.54, 1.53)	0.07 [0.03]	1.84 (1.10, 2.57)	**0.039** **[0.008]**	1.81 (1.22, 2.41)	29.56 ± 2.14
miR-22-3p	0.66 [0.55]	1.86 (0, 4.68) *	0.16 [0.075]	9.85 (0.07, 19.63)	0.13 [0.06]	6.16 (0.81, 11.5)	31.11 ± 3.75
miR-26b-5p	0.347 [0.225]	1.98 (0.38, 3.57)	**0.012** **[0.001]**	4.52 (2.45, 6.58)	**0.006** **[3.08 × 10^−4^]**	3.98 (2.44, 5.52)	29.29 ± 2.55
miR-29a-3p	0.347 [0.231]	1.53 (0.66, 2.4)	**0.027** **[0.005]**	3.29 (1.73, 4.85)	**0.041** **[0.011]**	2.05 (1.26, 2.85)	28.92 ± 2.94
miR-32-5p	0.56 [0.414]	0.71 (0.015, 1.413)	0.19 [0.095]	3.45 (0.557, 6.342)	0.33 [0.193]	1.81 (0.577, 3.041)	33.29 ± 2.36
miR-103a-3p	0.8 [0.72]	1.07 (0.68, 1.47)	**0.015** **[0.002]**	2.00 (1.39, 2.62)	**0.006** **[1.59 × 10^−4^]**	2.04 (1.53, 2.55)	28.24 ± 2.01
miR-106a-5p	0.32 [0.18]	2.3 (0.39, 4.21)	**0.041** **[0.01]**	4.12 (1.76, 6.47)	**0.01** **[0.001]**	4.83 (2.69, 6.96)	30.60 ± 2.28
miR-146a-5p	0.23 [0.12]	2.39 (0.61, 4.18)	**0.045** **[0.014]**	4.14 (1.67, 6.61)	**0.045** **[0.014]**	2.74 (1.37, 4.11)	32.34 ± 3.87
miR-148a-3p	0.58 [0.45]	0.78 (0.2, 1.36)	0.46 [0.32]	1.45 (0.55, 2.37)	0.76 [0.66]	0.9 (0.43, 1.36)	29.10 ± 2.24
miR-148b-3p	0.65 [0.52]	0.82 (0.25, 1.38)	0.33 [0.2]	1.59 (0.68, 2.5)	0.75 [0.63]	1.12 (0.62, 1.62)	31.85 ± 1.42
miR-155-5p	0.47 [0.33]	1.56 (0.4, 2.72)	0.12 [0.05]	3.45 (1.02, 5.89)	**0.048** **[0.016]**	2.45 (1.29, 3.62)	33.47 ± 1.22
miR-191-5p	0.93 [0.91]	1.03 (0.47, 1.59)	0.057 [0.02]	2.16 (1.19, 3.14)	**0.025** **[0.004]**	2.25 (1.42, 3.07)	27.74 ± 2.18
miR-484	0.16 [0.08]	0.7 (0.37, 1.04)	0.94 [0.94]	1.02 (0.61, 1.42)	0.84 [0.77]	1.05 (0.71, 1.39)	30.00 ± 1.14

FC—fold change value is expressed as median ASC-US/LSIL/HSIL vs. NILM, *p* value < 0.05 is considered as statistically significant, adj. *p* value is FDR adjusted *p* value, statistically significant values are highlighted in bold, ASC-US—atypical squamous cells of undetermined significance, LSIL—low-grade squamous intraepithelial lesion, HSIL—high-grade squamous intraepithelial lesion, NILM—negative for intraepithelial lesion or malignancy, SD—standard deviation * CI truncated at 0 due to model-based approximation on transformed scale. *p* value—unadjusted *p*-value for testing H_0_: FCH = 1. q value—Benjamini–Hochberg FDR-adjusted *p*-value.

**Table 2:** Altered miRNA expression associated with HPV infection.

miRNAs	HPV (*n* = 36)		ct HPV Negative (*n* = 30)
	*q Value* [*p Value*]	FC (95% CI)	(Mean ± SD)
miR-17-5p	0.15 [0.06]	1.37 (0.99, 1.75)	29.47 ± 2.55
miR-22-3p	0.15 [0.07]	2.65 (0.85, 4.44)	30.12 ± 3.58
miR-26b-5p	**0.012 [0.001]**	2.62 (1.72, 3.52)	28.78 ± 2.93
miR-29a-3p	0.25 [0.21]	1.3 (0.83, 1.77)	28.53 ± 2.68
miR-32-5p	0.07 [0.02]	1.77 (1.14, 2.40)	32.60 ± 3.01
miR-103a-3p	0.24 [0.15]	1.18 (0.93, 1.44)	27.96 ± 2.21
miR-106a-5p	0.07 [0.02]	1.82 (1.14, 2.51)	29.71 ± 2.46
miR-146a-5p	**0.042 [0.006]**	2.27 (1.38, 3.16)	32.19 ± 2.97
miR-148a-3p	0.25 [0.2]	1.4 (0, 0.34) *	29.30 ± 2.21
miR-148b-3p	0.17 [0.09]	1.52 (0.91, 2.12)	32.07 ± 1.62
miR-155-5p	0.82 [0.76]	1.07 (0.60, 1.54)	32.57 ± 2.00
miR-191-5p	0.25 [0.2]	1.24 (0.87, 1.61)	27.54 ± 2.19
miR-484	0.89 [0.89]	1.02 (0.73, 1.31)	30.55 ± 1.8

FC—fold change value is expressed as median of HPV-positive vs. HPV-negative sample, *p* value < 0.05 is considered as statistically significant, adj. *p* value is FDR adjusted *p* value, HPV—human papillomavirus, SD—standard deviation. * CI truncated at 0 due to model-based approximation on transformed scale. *p* value—unadjusted *p*-value for testing H_0_: FCH = 1. q value—Benjamini–Hochberg FDR-adjusted *p*-value.

**Table 3:** Expression levels of selected miRNAs in cervical biopsy samples across CIN grades.

miRNAs	CIN 1 (*n* = 4)		CIN 2 (*n* = 19)		CIN 3 (*n* = 2)		CIN 3—CIS (*n* = 16)		ct Biopsy Negative (*n* = 4)
	*q* Value [*p* Value]	FC (95% CI)	*q* Value [*p* Value]	FC (95% CI)	*q* Value [*p* Value]	FC (95% CI)	*q* Value [*p* Value]	FC (95% CI)	(Mean ± SD)
miR-17-5p	0.52 [0.27]	1.77 (0.38, 3.15)	0.25 [0.07]	1.49 (0.95, 2.02)	0.96 [0.92]	0.95 (0, 2.00) *	0.53 [0.30]	1.26 (0.76, 1.76)	27.36 ± 2.88
miR-22-3p	0.52 [0.28]	21.15 (0, 58.31) *	0.24 [0.05]	10.38 (0.94,19.82)	0.99 [0.967]	0.92 (0, 4.84) *	0.43 [0.16]	5.08 (0, 10.78) *	28.58 ± 4.21
miR-26b-5p	0.52 [0.26]	2.26 (0.04, 4.47)	**0.01** **[0.001]**	3.22 (1.98, 4.47)	0.96 [0.91]	0.89 (0, 2.86) *	0.25 [0.07]	1.94 (0.91, 2.97)	26.71 ± 3.63
miR-29a-3p	0.50 [0.24]	2.85 (0, 5.96) *	0.24 [0.04]	2.09 (1.04, 3.14)	0.95 [0.87]	0.89 (0, 2.27) *	0.41 [0.14]	1.69 (0.76, 2.62)	26.88 ± 1.87
miR-32-5p	0.71 [0.52]	2.33 (0, 6.45) *	0.44 [0.19]	2.32 (0.33, 4.31)	0.90 [0.78]	0.74 (0, 2.61) *	0.83 [0.67]	0.84 (0.10, 1.59)	30.98 ± 3.45
miR-103a-3p	0.28 [0.09]	2.24 (0.80, 3.69)	**0.005** **[2.9** ** × 10** **^−4^** **]**	2.42 (1.70, 3.13)	0.75 [0.58]	1.34 (0.11, 2.58)	**0.048** **[0.006]**	1.89 (1.28, 2.51)	26.44 ± 2.18
miR-106a-5p	0.60 [0.38]	1.995 (0, 4.27) *	**0.005** **[1.9** ** × 10** **^−4^** **]**	4.04 (2.56, 5.53)	0.94 [0.85]	0.81 (0, 2.86) *	0.44 [0.17]	1.72 (0.66, 2.78)	27.69 ± 3.36
miR-146a-5p	0.90 [0.77]	1.21 (0, 2.62) *	0.24 [0.05]	0.64 (0.29, 1.00)	0.6 [0.33]	0.55 (0, 1.48) *	0.63 [0.43]	1.3 (0.53, 2.07)	29.64 ± 2.28
miR-148a-3p	0.44 [0.18]	6.75 (0, 15.31) *	0.25 [0.07]	2.24 (0.90, 3.59)	0.86 [0.71]	0.75 (0, 2.10) *	0.25 [0.08]	2.35 (0.85, 3.85)	28.27 ± 2.50
miR-148b-3p	0.99 [0.99]	0.99 (0, 2.06) *	0.61 [0.4]	1.29 (0.6, 1.99)	0.50 [0.24]	0.53 (0, 1.33) *	0.53 [0.31]	1.49 (0.53, 2.46)	30.89 ± 2.09
miR-155-5p	0.63 [0.42]	2.6 (0, 6.65) *	0.64 [0.46]	0.8 (0.25, 1.35)	**0.004** **[7.7 × 10^−5^]**	0.21 (0, 0.54) *	0.25 [0.06]	0.59 (0.17, 1.02)	31.07 ± 2.02
miR-191-5p	0.50 [0.23]	2.53 (0, 5.08) *	0.11 [0.01]	2.44 (1.30, 3.57)	0.91 [0.80]	0.85 (0, 2.07) *	0.18 [0.03]	2.36 (1.16, 3.58)	26.04 ± 2.21
miR-484	0.57 [0.35]	1.65 (0.25, 3.05)	0.82 [0.65]	1.1 (0.67, 1.52)	**0.048** **[0.005]**	0.36 (0, 0.79) *	0.73 [0.55]	1.15 (0.66, 1.64)	29.27 ± 1.81

FC—fold change value is expressed as median of CIN1/CIN2/CIN3/Cin3-CIS vs. negative biopsy samples, *p* value < 0.05 is considered as statistically significant, adj. *p* value is FDR adjusted *p* value, CIN 1—cervical intraepithelial neoplasia grade 1, CIN 2—cervical intraepithelial neoplasia grade 2, CIN 3—cervical intraepithelial neoplasia grade 3, CIS—carcinoma in situ, SD—standard deviation. * CI truncated at 0 due to model-based approximation on transformed scale. *p* value—unadjusted *p*-value for testing H_0_: FCH = 1. q value—Benjamini–Hochberg FDR-adjusted *p*-value.

**Table 4:** Patients’ clinical data.

Demographics		NILM (*n* = 15)	ASC-US (*n* = 12)	LSIL (*n* = 19)	HSIL (*n* = 26)
Age	Median, Range	42, 29–69	42, 28–60	35, 19–53	37, 25–77
HPV status	Positive	1	8	13	20
	Negative	14	4	6	6
Treatment	LEEP + Fractional curettage	0	5	4	8
	LEEP + Endocervical curretage	0	6	13	17
	Cervical amputation	0	0	0	1
	In the follow-up	0	1	2	0
	No treatment	15	0	0	0
Biopsy result	Negative	0	1	0	3
	CIN 1	0	2	3	1
	CIN 2	0	3	8	11
	CIN 3	0	0	1	1
	CIN 3-CIS	0	3	5	8
	Unknown	15	3	2	2

CIN 1—cervical intraepithelial neoplasia grade 1, CIN 2—cervical intraepithelial neoplasia grade 2, CIN 3—cervical intraepithelial neoplasia grade 3, CIS—carcinoma in situ, NILM—negative for intraepithelial lesion or malignancy, ASC-US—atypical squamous cells of undetermined significance, LSIL—low-grade squamous intraepithelial lesion, HSIL—high-grade squamous intraepithelial lesion, LEEP—loop electrosurgical excision procedure, HPV—human papillomavirus.

**Table 5:** Characteristics of microRNAs included in the study.

miRBase ID	Assay Catalogue Number	miRNA Sequence
hsa-miR-16-5p	YP00205702	5′-UAGCAGCACGUAAAUAUUGGCG-3′
hsa-miR-21-5p	YP00204230	5′-UAGCUUAUCAGACUGAUGUUGA-3′
hsa-miR-103a-3p	YP00204063	5′-AGCAGCAUUGUACAGGGCUAUGA-3′
hsa-miR-155-5p	YP02119311	5′-UUAAUGCUAAUCGUGAUAGGGGUU-3′
hsa-miR-22-3p	YP00204606	5′-AAGCUGCCAGUUGAAGAACUGU-3′
hsa-miR-148a-3p	YP00205867	5′-UCAGUGCACUACAGAACUUUGU-3′
hsa-miR-191-5p	YP00204306	5′-CAACGGAAUCCCAAAAGCAGCUG-3′
hsa-miR-146a-5p	YP00204688	5′-UGAGAACUGAAUUCCAUGGGUU-3′
hsa-miR-26b-5p	YP00204172	5′-UUCAAGUAAUUCAGGAUAGGU-3′
hsa-miR-106a-5p	YP00204563	5′-AAAAGUGCUUACAGUGCAGGUAG-3′
hsa-miR-148b-3p	YP00204047	5′-UCAGUGCAUCACAGAACUUUGU-3′
hsa-miR-484	YP00205636	5′-UCAGGCUCAGUCCCCUCCCGAU-3′
hsa-miR-17-5p	YP02119304	5′-CAAAGUGCUUACAGUGCAGGUAG-3′
hsa-miR-34a-3p	YP00206061	5′-CAAUCAGCAAGUAUACUGCCCU-3′
hsa-miR-29a-3p	YP00204698	5′-UAGCACCAUCUGAAAUCGGUUA-3′
hsa-miR-143-5p	YP00204570	5′-GGUGCAGUGCUGCAUCUCUGGU-3′
hsa-miR-32-5p	YP00204792	5′-UAUUGCACAUUACUAAGUUGCA-3′
hsa-miR-423-3p	YP00204488	5′-AGCUCGGUCUGAGGCCCCUCAGU-3′
SNORD48	YP00203903	
UniSp6	YP00203954	

## Data Availability

The data is available upon request from the authors.

## Supplementary Materials

The following supporting information can be downloaded at: https://www.mdpi.com/article/10.3390/epigenomes10010001/s1, the signalling pathways, along with corresponding *p*-values and dysregulated genes, are listed in Supplementary File S1, raw qPCR data are attached in Supplementary File S2, R code is provided in Supplementary File S3.
